# Treatment of anal fistulas with Obsidian RFT^®^: just another autologous compound platelet-rich fibrin foam?

**DOI:** 10.1007/s10151-024-02968-6

**Published:** 2024-08-02

**Authors:** C. Dawoud, K. Girgis, A. Stift, F. Harpain, S. Riss

**Affiliations:** Division of Visceral Surgery, Department of General Surgery, Medical University, Währinger Gürtel 18-20, 1090 Vienna, Austria

**Keywords:** Anal fistula, Minimal invasive surgery, Platelet-rich fibrin foam, Obsidian

## Abstract

**Background:**

Sphincter-preserving techniques like autologous compound platelet-rich fibrin foam have gained popularity, offering potential for better functional outcomes in anal fistula treatment. The present study aimed to evaluate the efficacy and safety of Obsidian RFT^®^.

**Methods:**

The study conducted a retrospective analysis from January 2018 to December 2022 on patients who received anal fistula closure with Obsidian RTF^®^ at the Department of General Surgery, Medical University of Vienna. Clinical diagnosis, complemented by radiographic imaging, was employed to confirm inconclusive cases.

Demographic and fistula characteristics and postoperative data were collected from electronic records following STROCSS criteria.

**Results:**

Fifteen patients received Obsidian RFT® treatment for anal fistulas. We found no intra- and postoperative complications. The median hospital stay was 3 days. After a median follow-up of 32 months, a closure rate of 53.3% was detected.

Non-significant differences were observed in various variables, yet trends emerged, indicating associations between abscess presence and non-healing fistulas. A distinct age threshold (≥ 42.7 years) served as an indicator for an inability to achieve anal fistula cure.

**Conclusion:**

Obsidian RFT^®^ represents a safe, minimally invasive operative procedure. Approximately half the patients experienced healing, with better outcome in a younger population.

**Trial Registration:**

Ethical Approval number Medical University of Vienna (#1258/2018). This study was registered retrospectively in ClinicalTrials.gov (NCT06136325).

## Introduction

The incidence of anal fistulas varies widely, ranging from 0.1 to 0.3 per 1000 individuals in the general population, with higher prevalence rates in patients with inflammatory bowel disease [1].

The management of fistula disease has been challenging since ancient times, and recurrence of disease is a common complication after surgical closure. Traditional invasive treatment options aim to eliminate the fistula tract and to close the internal opening. Notably, such procedures carry the risk of damaging the anal sphincter complex and potentially lead to faecal incontinence symptoms [2, 3]. In recent years, sphincter-preserving techniques have gained popularity because of their potential to achieve better functional outcomes while effectively treating the fistula [4].

One sphincter-preserving technique in the treatment of anal fistulas is the use of autologous compound platelet-rich fibrin foam, a novel application of fibrin matrix [5]. Fibrin glue, composed of fibrinogen and thrombin, has been widely used in various surgical specialties for its adhesive properties and ability to promote tissue healing [6]. The autologous compound platelet-rich fibrin foam combines the benefits of fibrin glue with autologous blood, creating a more durable and resistant seal [7]. This recognition is attributed to its elevated growth factor concentration and its efficacy in coordinating essential processes in tissue regeneration, including cell proliferation, chemotaxis, cell differentiation and extracellular matrix synthesis [8–13].

However, its efficacy is not well studied in the literature. Pérez Lara et al. included 60 anal fistula patients who were treated with platelet-rich fibrin foam in a prospective longitudinal multicentre study and observed a success rate of 66.6% after a follow-up of 24 months [5].

Obsidian RFT^®^ represents a new product of platelet-rich fibrin foams, which has already shown success rates in additive healing and stabilization of colorectal anastomoses, reducing the insufficiency rate to up to 2.3% [14, 15].

Platelet-rich plasma (PRP) enables the liberation of multiple growth factors crucial for cellular proliferation and angiogenesis [14]. As a result, PRP initiates the activation of the platelet cascade, leading to a more abundant reserve of stable fibrinogen than that produced through the polymerization of autologous blood after the addition of exogenous thrombin. Specifically, platelet-rich fibrin (PRF) presents an alternative approach whereby the structure of the concentrate permits a gradual release of proteolytic growth factors.

The aim of our study was to analyse the efficacy of an autologous compound platelet-rich fibrin foam, Obsidian RFT®, in treating anal fistula.

## Methods

### Patients and data collection

A retrospective single-centre cohort study was performed between January 2018 and 2022. Fifteen patients (5 female), receiving an anal fistula closure with Obsidian RTF^®^, were included in the final analysis. Prior to the operation, anal fistulas were diagnosed based on clinical examination at the Department of General Surgery, Medical University of Vienna's outpatient clinic. An MRI was conducted to further specify the type of anal fistula tract. Anal fistula course was classified according to the Parks Classification [16]. Patients with inflammatory bowel disease were also included into the study. All fistulas were drained with a loose seton for at least 4–6 weeks before attempting surgical closure.

In our institution, Obsidian RFT is offered to patients with anatomically complex anal fistulas (high transsphincteric, suprasphincteric or extrasphincteric) and to patients with anatomically simple anal fistulas (low transsphincteric, intersphincteric or superficial) who are considered at high risk of faecal incontinence if a fistulotomy is performed. This includes females with anterior fistulas, some degree of associated faecal incontinence, previous anal surgeries or Crohn’s disease patients without clinical or endoscopic signs of rectal disease activity.

Fistula healing was defined as the closure of all treated external openings and no secretion after external finger compression without symptoms of the disease.

Demographic and anal fistula-related data were collected using the electronic medical chart system.

This work has been reported in line with the STROCSS criteria [20]. It received approval from the ethics committee of the Medical University of Vienna (#1258/2018) and was registered retrospectively in ClinicalTrials.gov (NCT06136325).

### Matrix preparation

**Fig. 1:**
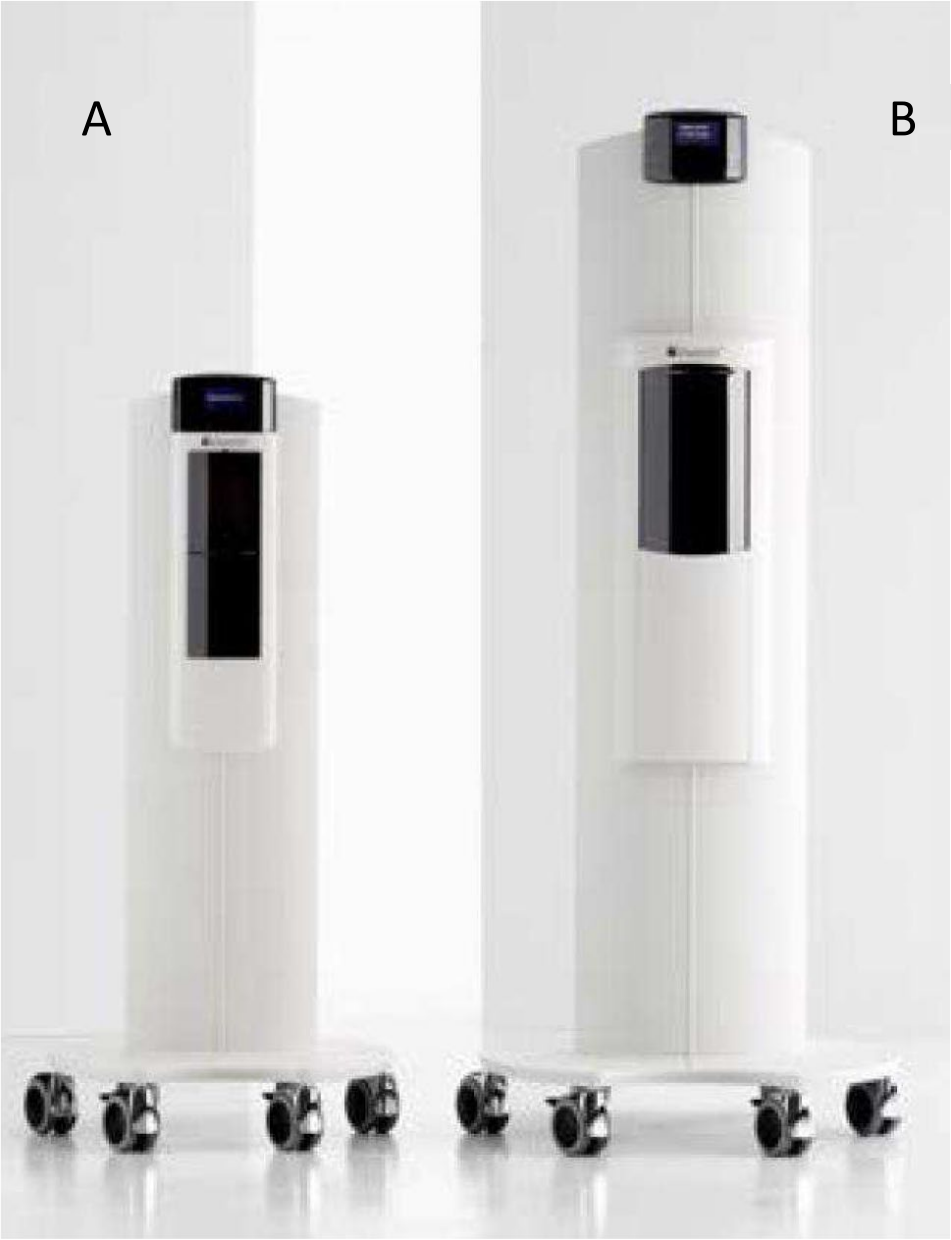
Two parts of the VIVOSTAD^®^ System. **A** Applicator unit. The applicator unit is used for the controlled application of the Obsidian RFT^®^ matrix to the area being treated. **B** Processing unit. The processing unit is used to prepare the patient's blood and to produce the bioactive Obsidian RFT^®^ matrix

The method for preparing the Obsidian RFT^®^ matrix has been previously described in a publication by Dauser et al. [15]. In summary, before surgery, each patient underwent the withdrawal of 120 ml of whole blood. The matrix was then prepared using 300 mg of tranexamic acid and processed through a fully automated Vivostat microprocessor-controlled system (Vivostat A/S, Alleroed, Denmark, see Fig. 1) [17]. The blood was heated to 36 °C and separated by centrifugation in the upper reservoir chamber of the processing unit. The resulting plasma was combined with Batroxobin, leading to the polymerization of acid-soluble fibrin 1. This process effectively removed excess fibrinogen and thrombocyte-depleted serum, leaving concentrated fibrin 1 and thrombocytes. To dissolve the available fibrin and create a stable clot matrix with high elasticity, tensile strength and crack resistance, the fibrin concentrate was mixed with sodium acetate buffer (pH 4). The resulting matrix, with up to tenfold thrombocytes compared to normal concentration, is embedded in a fibrin scaffold (Fig. 1). Around 5–6 ml Obsidian RFT^®^ is now available in a single-use application system specially designed for fistula surgery.

### Surgical technique

To begin with, the seton drainage was removed, and the fistula tract was cleaned and debrided using a special brush. Subsequently, the fistula was flushed with saline solution, and the inner fistula opening was exposed. A Z-suture was then performed to close the inner opening of the fistula. Afterwards, the Obsidian RFT^®^ endoscopic catheter was inserted from the distal outer opening of the fistula to the intraluminal proximal inner fistula ostium. Obsidian RFT^®^ was applied using the Vivostat "Jet no air" setting while slowly retracting the catheter. The external fistula opening was left open.

### Statistical analysis

Statistical analysis was performed using SPSS statistical software package (IBM SPSS Statistics for Mac, Version 22.0). Continuous variables are expressed as mean ± standard deviation or median and interquartile range, as appropriate; categorical variables are presented as numbers with percentage in brackets. Missing values are reported as unknown. Univariate analysis was performed using Student's *t* test to explore quantitative variables and chi-square test if they were dichotomous. A *p* value < 0.05 was considered to denote statistical significance.

## Results

Table 1 provides an overview of the patient demographics.

**Table 1:** Demographics and baseline characteristics

	*n* = 15
Demographics	
Age [years], median (range)	39.0 (21.0–61.0)
Female sex, *n* (%)	5 (33.3%)
BMI [kg/m^2^], median (range)	29.4 (20.5–37.9)
Clinical history	
Crohn associated fistula, *n* (%)	4 (26.7)
Cryptoglandular associated fistula, *n* (%)	11 (73.3)
Prior anal fistula surgery	
1 surgery, *n* (%)	7 (46.7)
2 surgeries, *n* (%)	4 (26.7)
≥ 3 surgeries, *n* (%)	4 (26.7)
Medication at surgery	
Immunosuppressive therapy, *n* (%)	4 (26.7)
Monoclonal antibodies, *n* (%)	3 (20.0)
Antimicrobial therapy, *n* (%)	15 (100)
Course of fistula tract	
Transsphincteric, *n* (%)	7 (46.7)
Intersphincteric, *n* (%)	4 (26.7)
Extrasphincteric, *n* (%)	2 (13.3)
Unclear, *n* (%)	2 (13.3)

The median duration of active symptomatic fistula until surgery was 14 weeks (IQR 7–20). All fistulas underwent Seton drainage prior to Obsidian RFT^®^ therapy.

Most of the fistulas showed a transsphincteric course (46.7%). The courses of fistulas are further outlined in Table 1.

### Surgical outcome

The median number of anal fistulas treated per patient was one with a maximum of five fistulas. All of the internal fistula openings were closed with a Z-suture (100%). The median operation time was 24 (range 6–55) min. The median length of hospital stay was 3 (range 2–5) days. We observed neither intra- nor postoperative complications.

The follow-up ranged from 18 to 57 (median 32) months.

We observed eight patients (53.3%) with complete fistula healing, demonstrated by a closure of the external opening and no symptoms of anal fistula like secretion by external compression.

The other patients, who showed no successful fistula closure, received a reapplication of a loose Seton drainage.

Due to incomplete MRI data, no systematic evaluation was performed.

**Fig. 2:**
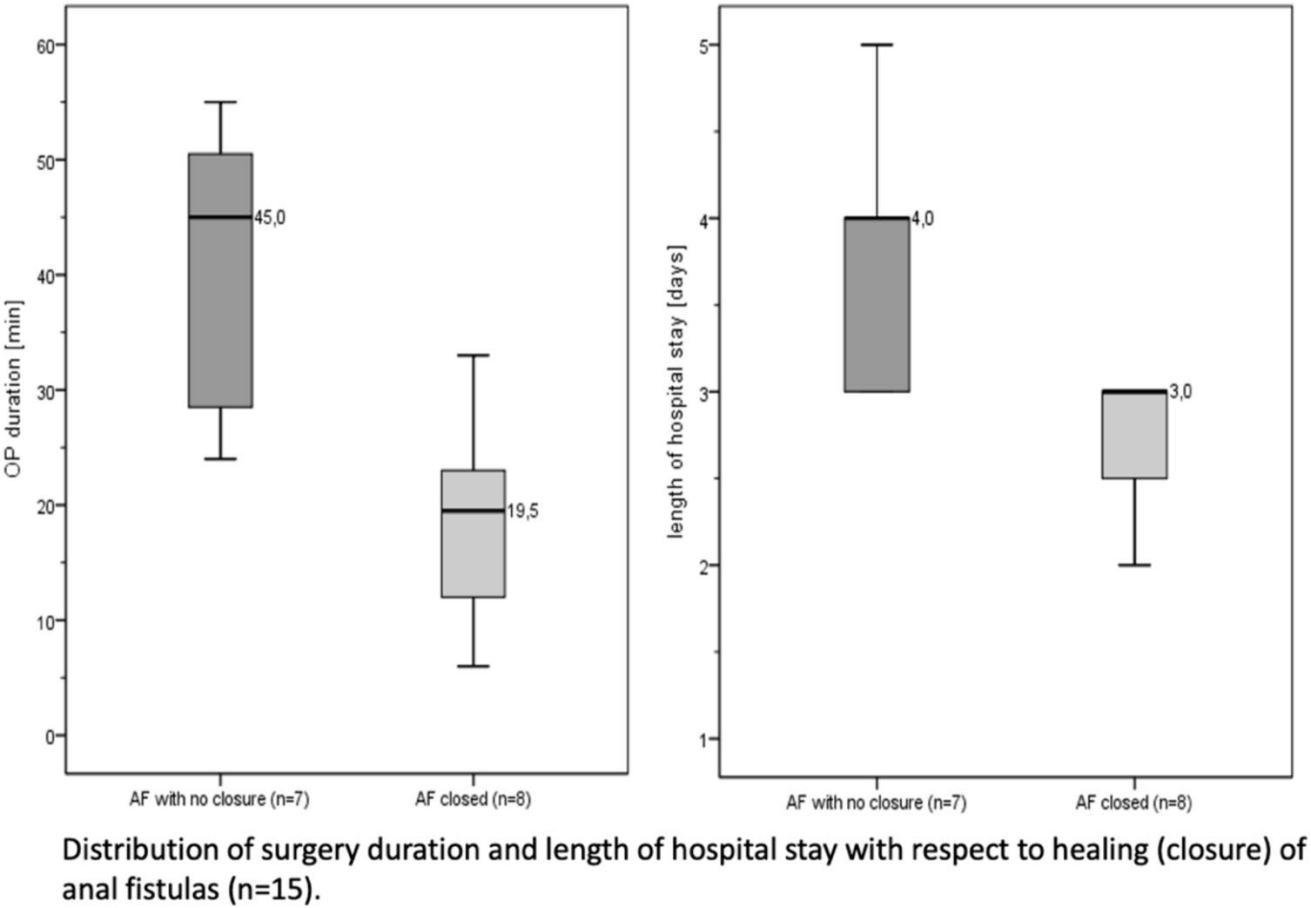
Distribution of surgery duration and length of hospital stay regarding healing (closure) of anal fistulas (*n* = 15)

Results of the univariate analysis are further outlined in Table 2. In case of an abscess, which was detected during surgery, a non-significant trend towards less successful fistula closure was observed (*p* = 0.077). Longer operation times and prolonged hospital stays were associated with significantly less favourable outcomes (*p* < 0.05), as outlined in Fig. 2.

**Table 2:** The distribution of categories of study-relevant variables and metric parameters in 15 patients with complete protocols is shown in the baseline characteristics

Parameters	Total (*N* = 15)	Healing		*p* value (effect *r*)
		No closure (*n* = 7)	Closed (*n* = 8)	
Crohn fistula	4 (26.7%)	1 (14.3%)	3 (37.5%)	> 0.569 (c.F.)
Cryptoglandular fistula	11 (73.3%)	6 (85.7%)	5 (62.5%)	
Single shot AB-prophylaxis				
None	2 (13.3%)	0	2 (25.0%)	0.386 (c.F.)
Metronidazol	4 (26.7%)	3 (42.9%)	1 (12.5%)	
Metronidazol and cefuroxim	9 (60.0%)	4 (57.1%)	5 (62.5%)	
Course of fistula				
Transsphincteric	7 (46.7%)	2 (28.6%)	5 (62.5%)	0.652 (c.F.)
Intersphincteric	4 (26.7%)	3 (42.9%)	1 (12.5%)	
Extrasphincteric	2 (13.3%)	1 (14.3%)	1 (12.5%)	
Unclear	2 (13.3%)	1 (14.3%)	1 (12.5%)	
Intraoperative abscess				
Yes	3 (20.0%)	3 (42.9%)	0	0.077° (c.F.)
No	12 (80.0%)	4 (57.1%)	8 (100%)	
Duration of surgery (min)				
Min–max	6–55	24–55	6–33	0.002**
* Md* (IQR)	24 (19.5; 39)	45 (28.5; 50.5)	19.5 (12; 23)	(0.76)
Length of hospital stay(days)				
Min–max	2–5	3–5	2–3	0.013*
* Md* (IQR)	3.0 (3.0; 3.5)	4.0 (3.0; 4.0)	3.0 (2.5; 3.0)	(0.64)
Postoperative AB treatment				
None	11 (73.3%)	4 (57.1%)	7 (87.5%)	0.282 (c.F.)
Yes	4 (26.7%)	3 (42.9%)	1 (12.5%)	

*AB* antibiotics

**p ≤ 0.01

*p ≤ 0.05

°*p* ≤ 0.10

**Fig. 3:**
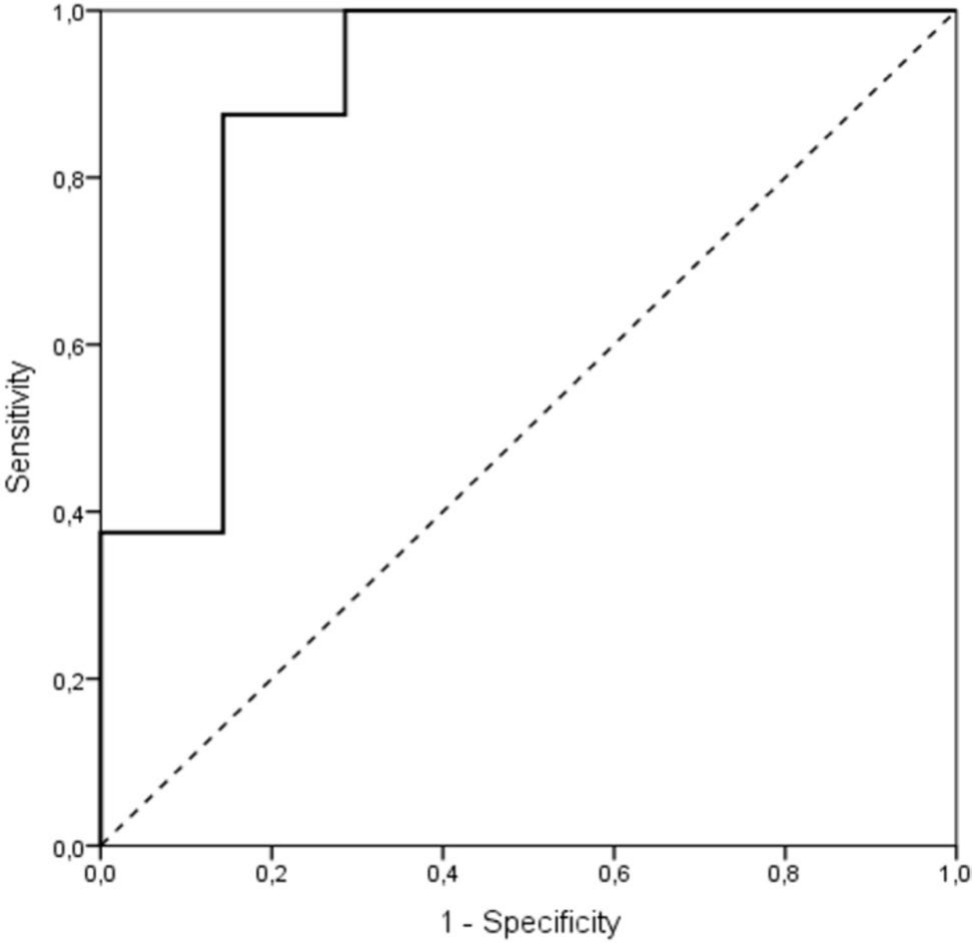
A ROC function of age on the criterion of not curing AF (*n* = 15)

Prognostic significance was evaluated for all univariate variables using receiver-operating characteristic (ROC) curves. Notably, only age was shown to be of predictive value. Table 3 depicts the outcomes of ROC analysis pertaining to the criterion of anal fistula cure. Alongside the area under the curve (AUC), the respective age thresholds (c) were established by maximizing the Youden's Index (YI). The findings related to these thresholds are presented, accompanied by sensitivity (Se) and specificity (Sp) values within the framework of the ROC analysis, along with their corresponding 95% confidence intervals (CI) for the AUC. The outcomes notably indicate that an age threshold of ≥ 42.7 years uniformly represents an optimal criterion for predicting the inability to achieve anal fistula cure. The ROC curves illustrating the discrimination potential of the age parameter for forecasting anal fistula cure, along with the corresponding markers for the optimal balance between sensitivity and 1-specificity, are shown in Fig. 3.

**Table 3:** The outcomes of the receiver-operating characteristic (ROC) analysis pertaining to the criterion of anal fistula cure

*n*	AUC	*SE*	*p* value	95% CI AUC		Sensitivity	Specificity	YI	Cut-off C
				LL	UL				
15	0.893	0.092	0.011	0.712	≤ 1.0	0.875	0.857	0.73	42.7 years

*AUC* area under the curve, *SE* standard error, *LL* lower limit, *UL* upper limit, *YI* Youden Index

## Discussion

The finding of our cohort study showed that in a difficult-to-treat patient population with complex perianal fistula, a 53.3% healing rate was achieved using Obsidian RFT^®^. Additionally, our data confirmed the safety of this therapy with no observed serious adverse events in the perioperative period.

Currently, no standard treatment exists in dealing with complex anal fistula. Several surgical techniques have been introduced in recent years, aiming to be effective, minimally invasive and sphincter preserving. Previous more invasive techniques, such as endoanal advancement flap repair, are still commonly conducted but do not guarantee healing in all patients. Additionally, impairment of anal incontinence could be observed in 10–35% of operated patients [2, 18].

Therefore, it is critical in the treatment of complicated perianal fistulas to develop innovative procedures and improve existing methods to optimize outcomes and minimize the impact on anal continence [19, 20].

The application of fibrin sealant for the management of postoperative enterocutaneous fistulas was initially documented in the 1980s [21]. Subsequently, fibrin glue was also implemented in the surgical management of anal fistula because of its easy use and the potential advantage of preservation of incontinence. Successful closure of the fistula showed a wide range between 14 and 86% [22–25]. Concurrently, platelet-rich fibrin, owing to its high concentration of growth factors and its capacity to orchestrate crucial processes in tissue regeneration, such as cell proliferation, chemotaxis (directed cell migration), cell differentiation and extracellular matrix synthesis, has gained further attention as a regenerative tissue modality with potentially better results [8–13].

In a prospective double-blind randomized study, De La Portilla et al. demonstrated that the therapeutic efficacies of two procedures, autologous platelet-rich plasma and autologous fibrin, were equivalent, with no observed adverse events. However, both procedures revealed recurrence rates of 33.3% and 31.3%, respectively [26].

Pérez Lara et al. reported on their prospective longitudinal multicentre study of anal fistula therapy with platelet-rich fibrin in 60 patients. A success rate of 66.6% was achieved after a 24-month follow-up [5]. In contrast to our study, patients with Crohn's disease were excluded from the study population.

Another study with only a small number (*n* = 10) of enrolled patients showed favourable results for anal fistula treatment with autologous platelet-rich plasma and platelet-rich fibrin glue [27]. Abdollahhi et al., who used autologous platelet-rich plasma (PRP) as well as platelet-rich fibrin glue (PRFG), showed a healing rate in 60% of patients. In contrast to other studies, 2 ml of PRP was injected into the tissue around the fistula (the depth of penetration at injection was 5–6 mm), and 4 ml of PRFG was mixed with 1 ml of thrombin and injected into the tract [27].

Van der Hagen et al. presented the therapeutic concept of injecting autologous platelet-rich plasma as an adjunct to a mucosal advancement flap in 10 patients. They observed a recurrence in only one patient [28].

Unlike the aforementioned studies, Perez et al. introduced a treatment approach involving the administration of platelet-rich fibrin into the fistula, omitting the closure of the internal orifice. This method was performed exclusively on an outpatient basis using anesthesia [29]. They reported a healing rate solely by in fistula application of 52.86% non-Crohn patients with an average of 1.92 sealant procedures.

Closure of the internal fistula opening is considered a logical healing approach to keep the fistula track clean in terms of preventing bacterial contamination. Controlled randomized clinical studies in the placebo group showed that the closure of the internal anal fistula opening alone can produce a healing value of approximately 36% in Crohn's patients [30].

In our results, a longer operation time and a prolonged hospital stay were associated with significantly less favourable outcomes, which may indicate that a shorter operation time reflects an easier procedure. Parameters indicating a more simple operation could not be determined as no differences in the complexity of the fistulas, experience of the surgeons or other factors were detected.

The analysis of our results also showed that patients < 42.7 years old had a better outcome in contrast to patients with of older age. It could be speculated that certain factors contribute to this finding. The number and activity of stem cells in the skin decrease with age, which can significantly hamper the skin's ability to regenerate [31]. Additionally, aging leads to a decline in the production of essential growth factors, contributing to slower wound healing processes. The structural integrity of the skin deteriorates, with the epidermal and dermal layers becoming thinner and less elastic, making the skin more susceptible to injury and slowing recovery [31]. Furthermore, chronic diseases such as diabetes and vascular disorders, which are more prevalent in older populations, impair blood flow and nutrient delivery to wound sites, exacerbating healing difficulties.

When interpreting the results of the present study several limitations need to be considered. The sample size of included patients is small. and due its retrospective study design, selection bias cannot be ruled out.

Although the inclusion of Crohn's patients leads to a higher heterogeneity of the treated population, it shows even more that a good cure rate is associated with Obsidian RFT despite more difficult systematic circumstances.

However, considering the lack of clinical therapeutic options for sphincter-preserving methods, we still believe that we can further provide important results to more clearly define the role of Osidian RFT^®^ in anal fistula treatment.

## Conclusion

Obsidian RFT^®^ introduces a novel and safe form of platelet-rich fibrin foams in the treatment of anal fistulas. Its efficacy is anticipated in approximately half of the patient cohort with better results in a younger population.

## Data Availability

The raw datasets generated during and/or analyzed during the current study are not publicly available due to the sensitive nature of the questions asked in this study but are available from the corresponding author on reasonable request.
